# Impact of a Nutrition Protocol on Vitamin D Supplementation in a Pediatric Intensive Care Unit: A Retrospective Cohort Study

**DOI:** 10.3390/clinpract15100186

**Published:** 2025-10-13

**Authors:** Maria Pérez Marin, Vivianne Chanez, Guillaume Maitre, Laurence Boillat, Frida Rizzati, Pauline Lauwers, Maria-Helena Perez

**Affiliations:** Pediatric Intensive Care Unit, Service of Paediatrics, Women-Mother-Children Department, Lausanne University Hospital, Rue Du Bugnon 21, 1011 Lausanne, Switzerland; vivianne.chanez@chuv.ch (V.C.); guillaume.maitre@chuv.ch (G.M.); laurence.boillat@chuv.ch (L.B.); frida.rizzati@chuv.ch (F.R.); marie-helene.perez@chuv.ch (M.-H.P.)

**Keywords:** vitamin D deficiency, vitamin D supplementation, nutrition protocol, pediatric intensive care unit

## Abstract

Background: Vitamin D deficiency (VDD) is highly prevalent in pediatric critically ill patients and is a potentially modifiable risk factor during critical illness. There are no established national or international recommendations for vitamin D supplementation in Pediatric Intensive Care Unit (PICU) patients. Objectives: This monocentric study aims to compare the practices regarding vitamin D supplementation before and after the introduction of a nutrition protocol (NP). Methods: We retrospectively analyzed vitamin D administration (time from PICU admission to initiation, amount of supplementation, accordance with existing guidelines) in children aged 0 to 16 who were admitted to the PICU of Lausanne University Hospital for more than 48 h the year before and the year after the introduction of a NP. Results: Vitamin D supplementation increased after NP introduction (95 IU per day more, *p* < 0.0001). More patients received vitamin D during their stay (95% after vs. 77% before, *p* < 0.0001). The dose adhered to NP recommendations for children under 12 and was higher for older children. According to Swiss guidelines for the general pediatric population, vitamin D supplementation was accurate in children under one year old before and after NP implementation. However, it was less than recommended for patients over one year old. Conclusions: The implementation of a NP significantly enhanced the scope of vitamin D supplementation. This study also highlights the practical limitations in meeting the recommended requirements with certain galenic formulations.

## 1. Introduction

Vitamin D is crucial in calcium-phosphate metabolism, bone mineralization, and many metabolic pathways modulating the immune system, cellular growth, and differentiation [1,2,3,4,5]. It is obtained through skin synthesis via UVB sunlight exposure and dietary sources. While definitions of deficiency vary, serum 25(OH)D levels below 50 nmol/L are widely considered deficient, and levels below 25–30 nmol/L are categorized as severe deficiency. These cutoffs are particularly relevant for bone health, where severe deficiency may lead to rickets and osteomalacia [5]. Some researchers propose that, considering vitamin D’s pleiotropic effects beyond bone health, serum levels of 25(OH)D should be >75 nmol/L, which would mean that most people worldwide could be considered to have vitamin D ‘insufficiency’ [6,7].

Strategies to improve vitamin D status in the general population include increased UV-B exposure, dietary intake, food fortification, supplementation, and weight loss [2,3]. Historically, 400 International Units (IU)/day was recommended based on the vitamin D content of one teaspoon of cod liver oil, which prevented rickets. Modern guidelines vary globally but generally agree that infants and at-risk populations require supplementation. A review by Bouillon noted that over 40 countries recommend 400 IU/day for infants, extending to toddlers and sunlight-deprived individuals, with doses ranging from 100 to 2000 IU/day [8].

In Switzerland, the Federal Office of Public Health (FOPH), responsible for national healthcare policy, recommends 400 IU/day for infants and 600 IU/day for at-risk children over one year, targeting a 25(OH)D level above 50 nmol/L [9,10]. The Swiss Society of Pediatrics (SSP) similarly recommends 400 IU/day from the second week of life through the first year [11].

In pediatric critically ill patients, VDD at admission is highly prevalent around the globe, with rates ranging from 25% to 84% [5]. Pediatric Intensive Care Unit (PICU) patients are more prone to hypovitaminosis D than the general pediatric population because of reduced endogenous production, restricted dietary intake, stress situations with increased vitamin D tissue consumption, decreased hepatic and renal hydroxylation, malabsorption, and critical illness-related interventions. VDD has been associated with increased mortality, illness severity, need for vasoactive agents, mechanical ventilation and infection. Therefore, VDD could represent a potentially modifiable risk factor regarding illness severity and clinical outcome during critical illness [5,12]. However, no national or international recommendations exist for vitamin D supplementation in PICU patients.

Enteral nutrition protocols (NPs) are recommended to improve the initiation of enteral nutrition, improve nutritional intake, and reduce adverse events in high-risk populations [13]. To address this gap, our PICU has implemented an evidence-based nutrition protocol (NP) since 2018, updated biennially in accordance with current guidelines. This study aims to assess whether our NP has improved vitamin D supplementation practices—specifically, the timing of initiation post-admission and the dosage provided. A secondary objective is to compare our supplementation practices with national recommendations before and after NP implementation. The NP is provided in the Supplementary Materials.

## 2. Materials and Methods

This retrospective monocentric cohort study was conducted at the Lausanne University Hospital, Switzerland. The 12-bed PICU is a mixed medical, cardiac, and surgical unit with approximately 450 admissions annually.

We retrospectively collected data on vitamin D doses administered to children aged 0 to 16 (admission age in our PICU) who were hospitalized for more than 48 h during the year before and the year after the introduction of a NP in the unit (implemented in July 2018). Patients with a length of stay <48 h were excluded. The study was approved by the local ethics committee (CER-VD project ID 2021-00872).

Physicians prescribe nutritional support, vitamins, and trace elements according to the NP recommendations, which were developed by the medical team and nutritionists of the unit. It recommends nutritional supplementation such as vitamins and trace elements (preferably enteral, as soon as possible, or parenteral when the enteral route is unavailable) for every child admitted to our PICU. As no standard of care for vitamin D supplementation has been established during or after pediatric critical illness, supplementation recommendations were extrapolated from national recommendations for the general pediatric population (FOPH). We use multivitamin complexes to simplify the administration and utilize the galenic forms available, providing the closest doses as recommended (e.g., drops for children under 12, pills for children over 12 years old). The recommended dose of vitamin D supplementation in the NP is: IV 110 IU per day for children <35 kg, 220 IU per day for children >35 kg; if enteral 444 IU per day for children <12 years old, 200 IU per day for children > 12 years old.

Eligible patients were identified through the PICU mixed register, which meets regulatory and ethical standards applicable to research involving human beings and has been approved by the local ethics committee (CER-VD AO_2021-00001) and the Operational Center for Biobanks and Registries (COB CHUV_2020_009_RM), which is the entity that supports investigators in the implementation of their projects involving the reuse of data and samples in compliance with the legal and ethical framework in our hospital. Data were collected from patients admitted to the PICU before and after implementing the NP and were exported from the Clinical Information System (Metavision, Imdsoft) and the Clinical Information System (Soarian) into an Excel file (coded data). We collected clinical and sociodemographic characteristics of the study population (gender, age, weight at admission, size at admission, length of stay, mortality Pediatric Index of Mortality (PIM) score); timing of introduction of enteral feeding; vitamin D contained in enteral feeding; and timing, amount and duration of vitamin D substitution (IU per day), including both intravenously (IV) and non-IV.

Statistical analysis was performed using Stata 16 program. All values are expressed as numbers (n) and percentages (%), as means and standard deviations for normally distributed data and as medians and interquartile ranges (IQRs) for non-normally distributed data. The Mann–Whitney U test and the Wilcoxon signed-rank test were used for continuous values, and the Pearson’s chi-squared test was used for categorical data. According to our analysis and previous nutritional studies in our unit and the literature, we expected a minimum increase of 10% in vitamin D supplementation (IU per patient per day) after applying the NP. To detect this effect, we estimated that 100 children would be needed in each group to provide the study with a power of 80% and a type one error of 0.05.

## 3. Results

Data were collected from 628 patients, including 296 admitted during the year prior to and 332 in the year following the implementation of the NP. There were no statistically significant differences between the two groups in terms of age, gender, weight, height, or length of stay. However, the PIM score was significantly higher in the pre-NP group (*p* = 0.0054; Table 1).

Total vitamin D administration, including both intravenous (IV) and non-IV supplementation as well as vitamin D from enteral feeds, significantly increased after the implementation of the NP. The mean total daily dose rose from 399 IU/day (pre-NP) to 481 IU/day (post-NP) (*p *= 0.0007). Supplemental vitamin D alone increased from 289 IU/day to 384 IU/day (*p* < 0.0001). The timing of vitamin D initiation remained unchanged after NP implementation, with a median of 40 h from admission in both groups (*p* = 0.9). However, the proportion of patients receiving vitamin D increased significantly, from 77% pre-NP to 95% post-NP (*p* < 0.0001), representing a relative increase of 23.4%. In the subgroup analysis of patients who did not receive vitamin D supplementation, there was no statistically significant difference in age (6.94 years before NP vs. 7.62 years after NP; *p* = 0.4463) or length of stay (3.75 days before NP vs. 2.96 days after NP; *p* = 0.1454).

IV vitamin D doses adhered closely to NP recommendations for all weight categories. Oral supplementation also aligned with NP targets in patients under 12 years. In contrast, patients older than 12 years received unintentionally double the NP-recommended dose, consistent with national FOPH guidelines (Table 2 and Table 3).

When compared with FOPH recommendations, vitamin D supplementation (excluding nutrition) was adequate in children under 1 year of age in both groups (*p =* 0.5852 pre-NP and *p =* 0.0928 post-NP). In children aged 1–3 years and those >3 years, supplementation remained below recommended levels but improved significantly after NP implementation in children >3 years (*p =* 0.0000) (Table 3).

Additional details are provided in the Supplementary Materials.

## 4. Discussion

To our knowledge, this is the first study to demonstrate improved vitamin D supplementation in critically ill children following the implementation of an NP. Given the high prevalence of VDD and its potentially modifiable impact on clinical outcomes in PICU patients, it is noteworthy that a minimal intervention can enhance vitamin D administration, enabling better adherence to national recommendations for the general population.

Our findings show that, after the NP was implemented, nearly all children admitted to our unit received vitamin D supplementation compared to 77% prior to its introduction. Since there were no significant differences in age or length of stay between the two groups, it is reasonable to infer that vitamin supplementation became more systematic following the NP, likely due to increased physician awareness of its importance.

In our unit, vitamin D supplementation was administered according to the local NP for all patients receiving it intravenously, and for patients under 12 years when given orally. However, after NP implementation, patients older than 12 years received an oral dose twice the amount recommended by the NP. One possible explanation is that most patients were given the multivitamin galenic formulation in drops, intended for children under 12 (which contains double the vitamin D), instead of the pill formulation designed for patients over 12. This higher dose more closely aligns with the national FOPH recommendations for the general population.

A secondary goal of the study was to compare our vitamin D supplementation practices with national recommendations before and after the implementation of the NP. A review of the existing literature revealed no specific national or international guidelines addressing vitamin D supplementation in PICU patients. The Society of Critical Care Medicine (SCCM) and the American Society for Parenteral and Enteral Nutrition (ASPEN) in 2017, and the European Society of Pediatric and Neonatal Intensive Care (ESPNIC) in 2020, respectively provided specific guidelines and clinical recommendations for nutrition in critically ill children. The first one does not provide recommendations for vitamin D substitution [14]. The second one declares insufficient evidence to recommend pharmaconutrition in PICU [13]. In 2023, the European Society for Clinical Nutrition and Metabolism (ESPEN) published a practical guideline for clinical nutrition in the intensive care unit. They mention that micronutrients should be provided daily with parenteral nutrition to enable substrate metabolism, and that 25(OH)D status can be determined in all patients considered at risk of vitamin D depletion or deficiency. However, they stated there is uncertainty regarding the dosing and timing of vitamin D administration [15]. Additionally, vitamin D recommendations for the general pediatric population vary considerably between countries. Consequently, we used the national FOPH recommendations for the general population as a reference point for comparison.

When comparing vitamin D supplementation in our PICU to the FOPH recommendations, only patients under one year of age received adequate supplementation both before and after the introduction of the NP. To explain these findings, we hypothesize that, prescribing physicians—most of whom are pediatricians—are more familiar with the SSP recommendations for the general population than with those of the FOPH. Consequently, they recognize the importance of administering vitamin D to infants under one year old but may be less aware of the guidelines for older children. They are certainly also aware of the essential role of vitamin D in early-life bone metabolism but are less informed regarding its extraskeletal properties at later ages.

In children older than one year, vitamin D supplementation remained below the FOPH recommendations, despite higher doses being administered after the implementation of the NP compared to before. This finding is unsurprising, given that the vitamin D dosage proposed in the NP for children over one year is lower than the national recommendation. When developing the NP, we considered the available galenic formulations and prioritized the use of multivitamin complexes to simplify administration. Our results have led to a revision of the NP dosages to improve supplementation rates in accordance with national FOPH guidelines for children over one year of age. Currently, a new multivitamin galenic formulation in milliliters is used in our unit, allowing the administration of 400 IU of vitamin D per day for children under one year old, and 600 IU per day for children over one. This highlights the practical challenges of meeting recommended vitamin D requirements using certain galenic forms.

To further improve supplementation in children over one, it would be beneficial for clinicians if the SSP and the FOPH harmonized and regularly updated their vitamin D recommendations. Additionally, the PICU medical team needs to recognize that critically ill children are at risk not only of macronutrient deficiencies but also of micronutrient deficiencies. Therefore, they should be considered at risk for hypovitaminosis D and receive appropriate vitamin D supplementation.

These observations highlight the importance of monitoring the correct implementation of a newly introduced protocol and of being able to update it based on monitoring results, emerging evidence and updated recommendations. They also emphasize the need to regularly update and standardize national protocols, as well as promote specialized nutritional knowledge and practices tailored to PICU patients.

This study has several limitations. First, its retrospective and single-center design introduces potential methodological biases. While the pre-post analysis offers valuable insights into the average changes observed during the year following the implementation of the NP, it does not allow us to assess the stability of these changes over time. Second, it would have been helpful to examine additional characteristics of the population that did not receive vitamin D supplementation before and after the NP implementation. For example, evaluating factors such as overall health status (e.g., malabsorption syndromes, renal or hepatic insufficiency, or hypoparathyroidism) could help clarify why some children were not supplemented. The higher PIM score observed in the pre-implementation group, for instance, may suggest that these patients were more severely ill, and that nutritional support was consequently deprioritized.

However, the strengths of our study include the large number of patients enrolled and the short interval between the two study groups, which minimizes the likelihood that other interventions in our PICU may have influenced the results.

It is important to note that we did not assess vitamin D status upon patient admission. As a result, the exact prevalence of VDD in our population remains unknown, and supplementation was not adjusted accordingly. This represents a potential area for improvement and may be worth exploring in future research.

## 5. Conclusions

In conclusion, we emphasize the importance of incorporating vitamin supplementation into the overall nutritional strategy for critically ill patients, ensuring that at least the recommended doses for the general population are administered. PICU patients should be regarded as a high-risk group, and efforts should be made to prevent further deterioration of their nutritional and vitamin status during hospitalization. As demonstrated in our unit, the implementation of an NP with specific recommendations can contribute significantly to achieving this objective.

## Figures and Tables

**Table 1:** Demographics of the two study groups.

	Before Nutrition Protocol (NP) (*n* = 296)	After NP (*n* = 332)	*p*-Value
Gender male/female, *n* (%)	170 (57.43)/126 (42.57)	174 (52.41)/158 (47.59)	0.2072
Age (years)	2.29 [0.72–7.28]	2.64 [0.48–7.12]	0.8380
Weight at admission (kg)	12 [7–21.75]	12 [6.15–20.2]	0.5020
Height at admission (m)	0.91 [0.68–1.18]	0.9 [0.64–1.17]	0.5341
Length of stay (days)	5.08 [3.53–8.93]	5.13 [3.13–8.12]	0.8310
Pediatric Index of Mortality (PIM score)	2.14 [1.02–4.41] *	1.40 [0.79–3.83] **	0.0054

* *n* = 286 (10 patients did not have a PIM score); ** *n* = 324 (8 patients did not have a PIM score). Values are expressed as *n* (%) for categorical variables and median [IQR] for continuous variables. The Mann–Whitney U test was used for continuous data and the Chi-square test for categorical variables.

**Table 2:** Vitamin D supplementation compared with nutrition protocol (NP) recommendations.

	Intravenous (IV) Vitamin D NP Recommendation (IU/day) *	IV Administered Vitamin D (IU/Day of Parenteral Nutrition) *	*p*-Value
Weight at admission ≤ 35 kg (*n* = 26) **	110	110 [109.99–110]	0.2402
Weight admission > 35 kg (*n* = 1) **	220	220 [220–220]	1.0000
	Non-IV vitamin D NP recommendation (IU/day) *	Non-IV administered vitamin D (IU/day of stay being fed) *	***p*-value**
Age ≤ 12 years old (*n* = 283) ***	444	484.36 [231.67–576.28]	0.2920
Age > 12 years old (*n* = 47) ***	200	407.27 [459.47–580.31]	0.0000

* IU/day refers to per patient. ** Patients post-NP who received IV vitamin D supplementation. *** Patients post-NP who received non-IV vitamin D supplementation, nutrition excluded. Values are presented as *n* for categorical variables and median [IQR] for continuous variables. The *p*-values were calculated with the Wilcoxon signed-rank test.

**Table 3:** Vitamin D supplementation compared with Swiss Federal Office of Public Health (FOPH) recommendations before and after NP implementation.

	FOPH Recommendation (IU/Day) *	Vitamin D Supplementation (IV and Non-IV) Before NP (IU/Day of Stay) *	*p*-Value
Age ≤ 1 year old (*n* = 91)	400	414.75 [280.11–496.44]	0.5852
1–3 years old (*n* = 74)	600	400.92 [210.26–568]	0.0000
Age > 3 years old (*n* = 131)	600	99.31 [0–313.02]	0.0000
	FOPH recommendation (IU/day) *	Vitamin D supplementation (IV and non-IV) after NP (IU/day of stay) *	***p*-value**
Age ≤ 1 year old (*n* = 121)	400	388.25 [201.13–522.75]	0.0928
1–3 years old (*n* = 51)	600	419.68 [273.10–520.16]	0.0000
Age > 3 years old (*n* = 160)	600	355.55 [149.33–497.86]	0.0000

* IU/day refers to per patient. Values are presented as *n* for categorical variables and median [IQR] for continuous variables. The *p*-values were calculated with the Wilcoxon signed-rank test.

## Data Availability

The data presented in this study are available upon request from the corresponding author due to ethical restrictions.

## Supplementary Materials

The following supporting information can be downloaded at https://www.mdpi.com/article/10.3390/clinpract15100186/s1, Nutrition protocol: Protocole d’alimentation et suivi du transit 2018. Nutrition protocol: Protocole d’alimentation et suivi du transit 2021. Results and statistical tests.

## Abbreviations

The following abbreviations are used in this manuscript:

VDD	Vitamin D Deficiency
NP	Nutrition Protocol
PICU	Pediatric Intensive Care Unit
FOPH	Federal Office of Public Health
SSP	Swiss Society of Pediatrics
IV	Intravenously
PIM	Pediatric Index of Mortality
