# Supplementary material for: Impact of a Nutrition Protocol on Vitamin D Supplementation in a Pediatric Intensive Care Unit: A Retrospective Cohort Study
Source: Clin Pract. 2025 Oct 13;15(10):186. doi: 10.3390/clinpract15100186 (PMC12563859; doi:10.3390/clinpract15100186)
Supplement: Supplementary file 1 [file clinpract-15-00186-s001.zip › NP 2018 En.pdf]

Pediatric Intensive Care Unit: Enteral Feeding and Bowel Transit Monitoring Protocol

---

1. PURPOSE

This protocol outlines the steps for initiating and increasing feeding, selecting appropriate nutritional solutions, identifying major contraindications, and monitoring and stimulating bowel transit in critically ill pediatric patients.

2. SCOPE

Applicable to all medical, nursing, and assistant staff in the Pediatric Intensive Care Unit (PICU).

3. DEFINITIONS

- CEF: Continuous enteral feeding
- NG: Nasogastric
- ND: Nasoduodenal
- NJ: Nasojejunal
- PEG: Percutaneous Endoscopic Gastrostomy
- TPN: Total Parenteral Nutrition
- PPN: Partial Parenteral Nutrition
- ECCC: Extracorporeal circulation

4. RESPONSIBILITY

The medical and nursing staff are responsible for implementing this protocol.

5. DOCUMENTS AND REFERENCES

References to other protocols of the unit

6. INTRODUCTION AND GENERAL RULES

Nutrition in critically ill children is a key research and clinical focus in the PICU.

Enteral feeding should be initiated as early as possible (within the first 24 hours) in the absence of contraindications and increased gradually according to the child's risk factors and tolerance.

The longer the introduction of feeding is delayed, the poorer the tolerance and the higher the protein-calorie deficit. Both undernutrition and overnutrition increase the risk of morbidity and mortality in children.

Assessment of the child's nutritional status upon admission is essential to determine the need to initiate feeding, the need for vitamin and trace element supplementation, and to define caloric targets.

Body weight must be monitored rigorously throughout hospitalization as soon as the clinical condition allows it. The frequency of weighing should be assessed on a case-by-case basis.

Enteral feeding is generally introduced via CEF through a NG or ND tube, and less commonly via NJ tube or PEG.

In the event of failure of NG feeding or difficulties in placing an ND tube, ND placement should be considered promptly (within 48 hours) by the pediatric gastroenterology team.

Feeding should be transitioned to intermittent boluses as soon as the child's clinical condition improves, ensuring that nutritional requirements remain met—for example, by maintaining CEF during the night.

In cases of enteral feeding failure (after 72–96 hours), TPN or PPN must be initiated to prevent a significant protein-calorie deficit. If the CEF is insufficient to meet protein needs, protein supplementation (such as IV proteins or PPN) should be considered. Primary TPN remains an exception in the PICU, except in situations where enteral feeding is not feasible (e.g., chylothorax, major digestive surgery).

Bowel transit must be closely monitored, particularly during deep analgesia-sedation, which may cause pseudo-obstruction of the bowel, potentially leading to respiratory complications that prevent extubation or necessitate re-intubation, as well as significant infectious risks. Preventive laxative therapy should be considered during heavy sedation (no later than 72 hours).

Note: Laxatives can interfere with the absorption of certain medications. Care must be taken not to administer them simultaneously.

This document deliberately does not include dosing information. For all dosages, indications, contraindications, preparations, compatibilities, etc., please refer to the medication file of the unit.

7. INITIATION, INCREASE, AND MONITORING OF FEEDING

Enteral feeding should be initiated early in critically ill children, ideally within the first 24 hours of hospitalization, unless contraindicated:

- Digestive problems as the reason for PICU admission (e.g., post-digestive surgery; observe fasting instructions prescribed by the operator)

- First night post-cardiac surgery (light meals and fluids allowed in extubated children after a short duration of cardiopulmonary bypass)
- Low cardiac output (risk of mesenteric ischemia and necrotizing enterocolitis), regardless of etiology (cardiac, septic, etc.)

The rate of enteral feeding should be increased progressively, based on the patient's tolerance and clinical condition, in order to reach the energy target by day 4 of hospitalization.

1. <u>Neonates</u>: Feeding should be introduced according to neonatal guidelines, starting at a maximum of 20 ml/kg/day, with a daily increase of up to 20 ml/kg/day.

   Example: A neonate weighing 3.6 kg: 20 ml/kg = 72 ml/day → maximum continuous enteral rate of 3 ml/h.

   In cases of particular risk (e.g., hemodynamic instability, risk of mesenteric ischemia), such as during the reinitiation of feeding after cardiac surgery, AEDC should be initiated at 1 mL/h and increased by 1 mL/h every 24 hours to assess gastrointestinal tolerance initially. The increase in enteral feeding (frequency and volume) will depend on clinical evolution.

2. <u>Children under 10 kg</u>: Enteral feeding is introduced at a maximum rate of 1 ml/kg/h and increased every 4 to 24 hours by 1 ml/kg/h according to gastrointestinal tolerance and clinical condition.

   Example: Infant weighing 7 kg → initial maximum rate 7 ml/h.

3. <u>Children over 10 kg:</u> Enteral feeding is introduced at 5–10 ml/h and increased by 5–10 ml/h every 4 to 24 hours based on tolerance and clinical condition.

   Example: Child weighing 40 kg → start at 10 ml/h, increasing by 10 ml/h every 4–8 hours until reaching 40–50 ml/h initially, depending on available fluid intake.

   Note: These intakes alone do not meet the energy requirements for a 40 kg intubated and ventilated patient. Intravenous glucose infusions provide the necessary additional calories. Protein supplementation should be considered if required.

Energy and protein intakes received by patients—automatically calculated in the PDMS MetaVision®—must be checked daily. The energy contribution from IV glucose, which may be significant, especially in younger children, should be included in calculations. It is essential to ensure that each day's energy and protein intake meets the patient's requirements by calculating the energy and protein balance. The cumulative balance over the hospitalization period should also be monitored.

8. CALORIC AND PROTEIN TARGETS

The energy requirements of critically ill children are lower compared to those of healthy children, primarily due to the effects of mechanical ventilation, sedation, analgesia, and lack of physical activity. When a child is ventilated, sedated, and receiving analgesia/sedation, the energy target should not exceed their resting energy expenditure, which can be estimated using the Schofield predictive equation or according to the following average values:

Table 1: Recommended energy targets for critically ill children by age (kcal/kg/day)

	0–6 months	7-12 months	1–3 years	4–8 years	9–18 years
Energy target (kcal/kg/day)	58	62	58	46	Schofield Equation (weight/height)

Schofield Equation for Resting Energy Expenditure (kcal/day)

Girls
- 0–2 years: 16.252 × P + 1023.2 × (T/100) − 413.5
- 3–9 years: 16.97 × P + 1.618 × T + 371.2
- 10–18 years: 8.365 × P + 4.65 × T + 200

Boys
- 0–2 years: 0.167 × P + 1517.4 × (T/100) − 617.6
- 3–9 years: 19.6 × P + 1.033 × T + 414.9
- 10–18 years: 16.25 × P + 1.372 × T + 515.5

P = weight in kg; T = height in cm; results in kcal/day

When the child is stable and in the recovery phase (post-PICU or chronic patients), the energy target should also consider physical activity, growth, and cumulative energy debt. Resting energy expenditure can be increased by 20–50% depending on the situation, particularly if the child is active.

In contrast to energy requirements, protein requirements in critically ill children are elevated due to increased urinary nitrogen loss. The recommended intake to maintain nitrogen balance is a minimum of 1.5 g/kg/day, and may be higher in children over 4 years of age.

Caloric and protein targets must be entered into MetaVision® to allow daily monitoring of the child's nutritional status.

9. INDICATIONS FOR STOPPING FEEDING

Nutrition should only be interrupted in cases of acute deterioration of the patient:

- Hemodynamic: risk of low mesenteric blood flow
- Respiratory: risk of intubation or re-intubation
- Digestive: suspicion of ileus or necrotizing enterocolitis
- Neurological: impaired consciousness with risk of bronchial aspiration

It is also essential to stop nutrition prior to elective surgery. The timing for stopping nutrition is defined in Procedure SIP-PRO-0017 / "*Stopping Nutrition for Anesthesia Procedures*."

When the patient presents gastric stasis with high residua, defined as >4 ml/kg every 4 hours (for example, due to pyloric spasm or decreased gastrointestinal motility in the context of opioid treatment), the following measures should be considered:

- Continue CEF even if the rate cannot be increased as desired/prescribed.
- Consider placing a nasoduodenal tube (if necessary, under the guidance of gastroenterology specialists) if the patient is initially receiving gastric feeding.
- Adjust or change the prokinetic medications being used (see section 11).

10. FEEDING SOLUTIONS SELECTION

The choice of feeding solutions depends on the child's age, weight, and clinical indication.

Due to transit issues (constipation and diarrhea) in the unit, all selected nutritional products contain fiber whenever possible.

1. Up to 1 year of age:

    - Breast milk or infant formula (see formulas available in the institution), including milk brought by parents.

    - Infatrini®:
        - Indications: Malnourished infants, infants at risk of malnutrition, those with growth delay, increased energy needs, and/or fluid restriction; for infants from birth up to 9 kg or up to 18 months of age.
        - Composition: 1 kcal/ml (per 100 ml: 101 kcal, 2.6 g protein, 10.3 g carbohydrates, 5.4 g fat, 0.6 g fiber, GOS/FOS, 305 mOsm/l)

2. From 1 year of age:

- Nutrini Multifibres®:
    - Indications: For children aged 1–6 years or weighing 8–20 kg. Gluten- and lactose-free. Not suitable for infants, patients on a fiber-free diet, or patients with galactosemia.
    - Composition: 1 kcal/ml (per 100 ml: 101 kcal, 2.5 g protein, 12.5 g carbohydrates, 4.4 g fat, 0.8 g fiber, 205 mOsm/l)

3. From 6 years of age:

    - Isosource Fibres®:
        - Indications: Standard physiological nutrition for patients requiring long-term enteral feeding who are predisposed to constipation.
        - Composition: 1 kcal/ml (per 100 ml: 103 kcal, 3.9 g protein, 13.5 g carbohydrates, 3.4 g fat, 1.5 g fiber, 266 mOsm/l)

Other nutritional solutions may be used if clinically indicated, following specialist consultation and approval by the attending medical consultant.

11. VITAMINS AND TRACE ELEMENTS

A supplement of vitamins and trace elements should be provided as long as the caloric target has not been reached.

In the PICU, all patients receive a vitamin supplement (intravenous: Multivitamins Cernevit®, enteral: Multibionta® or Supradyn®, depending on age), trace elements, and cholecalciferol (vitamin D).

Trace elements dosage: 1 ml/kg, maximum 10 ml/day IV, 2 ml/kg, maximum 20 ml/day if enteral. Exception: if Supradyn® is administered enterally, additional trace elements are not needed, as it already contains them.

Vitamin D and zinc status should be checked in at-risk children, and zinc supplementation should be provided to those who are deficient.

In cases of renal insufficiency requiring peritoneal dialysis, caution is needed due to the potential accumulation of vitamins and trace elements.

12. PROKINETICS

Prokinetic treatments can be increased in dose or frequency as needed (see the medication file).

First-line prokinetic: Domperidone is used as the primary prokinetic in the PICU.

Second-line prokinetic: Metoclopramide is used if feeding with domperidone fails after 48 hours. Metoclopramide is particularly useful in cases of suspected pyloric spasm.

Third-line prokinetic: Erythromycin may be used to stimulate gastrointestinal motility in cases of subileus associated with opioid use.

13. BOWEL TRANSIT MONITORING AND LAXATIVE TREATMENT

A laxative treatment should be initiated no later than the third day without stool, and, if possible, after introducing enteral feeding that stimulates intestinal motility. It is not necessary to wait for full enteral feeding before starting laxatives.

Opioids have a strong paralytic and drying effect on the entire digestive tract. In our experience, the combination of opioids and clonidine often causes persistent constipation, sometimes requiring the use of multiple laxatives or a combination of laxatives and disimpaction treatments (glycerin suppositories, enemas).

For all patients, especially those under significant analgesia-sedation or the opioids-clonidine combination, daily monitoring of bowel movements is essential.

Factors that can cause or worsen constipation should be identified and, as far as possible, corrected, including dehydration, immobility, a low-fiber diet, hypercalcemia, hypokalemia, and certain medications (opioids, urinary spasmolytics, anticalciques, H1 antihistamines, neuroleptics, tricyclic antidepressants, iron, and calcium).

For children known to have chronic constipation or who are chronically treated with laxatives, it is recommended to resume their usual treatment as soon as possible, provided there are no contraindications.

In the PICU, the first-line laxative is macrogol (Movicol®). Its use (dose, number of sachets, frequency) is detailed in:

Utilisation des laxatifs à l'hôpital. Bulletin d'information CPM N°1-2012 http://tribu.intranet.chuv/content-09.09.2016_10_18.pdf

Paraffin oil and picosulfate can be added in cases of refractory constipation in children aged 1 year and 2 years, respectively.

Warning for paraffin oil: risk of bronchoaspiration; contraindicated in dysphagia, severe gastroparesis, significant gastroesophageal reflux, or neurological disorders.

Lactulose (Duphalac®, Gatinar®) has been discontinued due to abdominal bloating and related respiratory complications.

Mucilages are contraindicated in patients on opioids due to the risk of fecal impaction.

DISIMPACTION:

For disimpaction, glycerin suppositories (Bulboid) are used and, when necessary, combined with osmotic enemas using 0.9% NaCl with 10% glycerin at 10 ml/kg, repeated as needed. Enema volumes can be increased to 20 ml/kg upon medical advice.

14. VALIDATION

Version No.	Date	Prepared/Modified by	Validated by	Validation Date
1.0	25.06.2018	Marie-Hélène Perez (Doctor), Corinne Jotterand (Dietitian), Clémence Moulet (Dietitian), Jacques Cotting (Doctor)	Certification Working Group	17.07.2018
