# Supplementary material for: Impact of a Nutrition Protocol on Vitamin D Supplementation in a Pediatric Intensive Care Unit: A Retrospective Cohort Study
Source: Clin Pract. 2025 Oct 13;15(10):186. doi: 10.3390/clinpract15100186 (PMC12563859; doi:10.3390/clinpract15100186)
Supplement: Supplementary file 1 [file clinpract-15-00186-s001.zip › NP 2018.pdf]

Protocole d’alimentation et suivi du transit

1. OBJET

Cette procédure décrit comment introduire et augmenter l’alimentation, quels produits choisir, quelles sont les contre-indications majeures à l’alimentation et comment surveiller et stimuler le transit intestinal aux SIP.

2. DOMAINE D’APPLICATION

Le personnel médical et infirmier ainsi que les ASSC des SIP.

3. DÉFINITIONS

AEDC : Alimentation entérale à débit continu
SNG : sonde nasogastrique
SND : sonde nasoduodénale
SNJ : sonde nasojéjunale
PEG : « Percutaneous Endoscopic Gastrostomy » : gastrostomie endoscopique percutanée
TPN : « Total Parenteral Nutrition » : nutrition parentérale totale
PPN : « Partial Parenteral Nutrition »: nutrition parentérale partielle
CEC : circulation extracorporelle

4. RESPONSABILITÉS

L’application de cette procédure est sous la responsabilité des cadres médico-infirmiers.

5. DOCUMENTS ET TEXTES DE RÉFÉRENCES

Fichier médicaments.

L:\SIP\DOCUMENTS_SIP\01-SOINS\Digestif :

- SIP-PRO-0017_Arrêt de l'alimentation lors d'une procédure d'anesthésie_mise à jeun
- Produits pour alimentation par sonde.pdf – Raccourci
- Raccourci vers Laits_nourrissonsv2
- Raccourci vers Logimen_proalim_minimo[1].pdf

Utilisation des laxatifs à l’hôpital. Bulletin d’information CPM N°1-2012 http://tribu.intranet.chuv/content-09.09.2016_10_18.pdf

6. PREAMBULE ET REGLES GENERALES

Aux SIP, la nutrition de l’enfant gravement malade est un **axe de recherche** depuis de nombreuses années.

Protocole d'alimentation et suivi du transit

L'alimentation entérale doit être débutée dès que possible (dans les premières 24h) en l'absence de contre-indications et augmentée en fonction des risques et de la tolérance de l'enfant.
Plus on tarde à introduire l'alimentation, plus celle-ci sera mal tolérée et plus le déficit protéino-calorique sera élevé ! Une sous-nutrition et une surnutrition vont toutes deux augmenter les risques de morbidité et de mortalité chez l'enfant.

L'évaluation de l'état nutritionnel de l'enfant à l'entrée est indispensable afin de juger de l'urgence à introduire une alimentation et un supplément en vitamines et en oligo-éléments et de définir les cibles caloriques.
Le poids doit être rigoureusement suivi durant toute l'hospitalisation dès que la situation clinique le permet. La fréquence de la pesée doit être évaluée au cas par cas.

L'alimentation entérale est introduite en règle générale avec un débit continu (AEDC) par SNG ou SND, et plus rarement par SNJ ou PEG.
En cas d'échec d'alimentation par SNG et de problèmes de pose d'une SND, il faut rapidement (dans les 48h) envisager une pose de SND par l'équipe de gastro-entérologie pédiatrique.
L'alimentation est fractionnée dès que la situation clinique de l'enfant le permet et en s'assurant que ses besoins nutritionnels soient toujours couverts, par exemple en maintenant une AEDC nocturne.

En cas d'échec de l'alimentation entérale (après 72h-96h), une alimentation parentérale totale ou partielle (TPN, PPN) doit être introduite pour ne pas mettre l'enfant en déficit protéino-calorique important. Si l'AEDC est loin de couvrir les besoins en protéines, une solution de protéines (Aliped, PPN) doit être envisagée. La TPN en première intention reste aux SIP une exception sauf dans les situations où une alimentation entérale n'est pas envisageable (chylothorax, chirurgie digestive lourde, etc.)

Le transit doit être rigoureusement surveillé, notamment lors d'analgo-sédations profondes qui peuvent mener à des pseudo-obstructions intestinales entraînant potentiellement des problèmes respiratoires empêchant une extubation ou motivant une ré-intubation ainsi que des risques infectieux majeurs. Un **traitement laxatif préventif** doit être envisagé lors de sédations lourdes (au plus tard après 72h).

NB : les laxatifs peuvent perturber l'absorption de certains médicaments. Attention à ne pas les administrer en même temps !

Ce document ne comporte volontairement aucune posologie. Pour toutes les posologies, indications, contre-indications, préparations, compatibilités, etc., veuillez-vous référer au fichier médicaments.

Protocole d'alimentation et suivi du transit

7. INTRODUCTION DE L'ALIMENTATION, AUGMENTATION ET SUIVI

L'alimentation entérale doit être débutée précocement chez l'enfant sévèrement agressé, si possible dans les premières 24h d'hospitalisation à moins qu'il n'y ait une contre-indication :

- Problèmes digestifs comme motif d'hospitalisation aux SIP (par exemple post chirurgie digestive, respecter les mises à jeun de l'opérateur)
- Première nuit post chirurgie cardiaque (boissons et repas léger autorisé chez l'enfant extubé après courte durée de CEC)
- Bas débit cardiaque (risque d'ischémie mésentérique et d'entérocolite nécrosante) quelle que soit l'origine (cardiaque, septique, etc…)

L'augmentation du débit de l'alimentation entérale sera progressive, en fonction de la tolérance du patient et de sa situation clinique, de manière à atteindre la cible énergétique à J4 de l'hospitalisation.

1. Chez le **nouveau-né**, l'introduction de l'alimentation se fait selon les directives de néonatologie, c'est-à-dire débuter avec 20 ml/kg/jour maximum et augmentation quotidienne de 20 ml/kg/jour maximum :
   Exemple : nouveau-né de 3,6kgs : 20 ml/kg =72 ml/jour => débit entéral maximal de 3 ml/h.
   En cas de risques particuliers ***(instabilité hémodynamique en particulier, risque d'ischémie mésentérique)*** *comme lors d'une reprise de l'alimentation après une chirurgie cardiaque, l'AEDC commencera à 1 ml/h et sera augmentée de 1 ml/h chaque 24h au début afin de tester la tolérance digestive. L'augmentation du débit de nutrition entérale (fréquence et quantité) dépendra de l'évolution clinique.*

2. Chez **l'enfant de moins de 10kgs**, l'alimentation entérale est introduite au débit maximal de 1 ml/kg/heure et augmentée toutes les 4 à 24h de 1 ml/kg/h en fonction de la tolérance digestive de l'enfant et de la situation clinique.
   Exemple : nourrisson de 7 kg => 7 ml/heure comme débit maximal initialement

3. Chez **l'enfant de plus de 10kgs**, l'alimentation entérale est introduite à 5-10 ml/h et augmentée de 5-10 ml/h toutes les 4 à 24h en fonction de la tolérance digestive de l'enfant et de la situation clinique.
   Exemple : Enfant de 40kgs : 10 ml/h augmenter de 10 ml/h chaque 4-8 h jusqu'à 40-50 ml/h initialement, en fonction des apports liquidiens à disposition.
   A noter que ces apports ne permettent pas de couvrir les cibles énergétiques pour un patient de 40kgs intubé et ventilé. Néanmoins, les perfusions glucosées apportent le supplément nécessaire. Si nécessaire un supplément de protéines (Aliped) doit être envisagé.

Les apports énergétiques et protéiques reçus par les patients – calculés automatiquement sur MetaVision - doivent être vérifiés quotidiennement. L'apport énergétique fourni par le glucose en iv, qui peut représenter un apport élevé surtout chez les plus jeunes, est pris en compte dans les calculs. Il est indispensable de s'assurer au quotidien de l'adéquation entre les apports en énergie et protéines reçus par les patients et leurs besoins, en calculant la balance énergétique et protéique. La balance cumulée sur le séjour est également à vérifier.

Protocole d'alimentation et suivi du transit

8. CIBLES CALORIQUES ET PROTEIQUES

Les besoins énergétiques de l'enfant sévèrement agressé sont bas en comparaison à ceux de l'enfant en santé, notamment de par les effets de la ventilation mécanique, la sédation, l'analgésie et l'absence d'activité physique. Lorsque l'enfant est ventilé, sédaté et analgésié, la cible énergétique ne devrait pas dépasser sa dépense énergétique de repos qui peut être estimée avec l'équation de prédiction de Schofield ou selon les valeurs moyennes suivantes :

	0-6 mois	7-12 mois	1-3 ans	4-8 ans	9-18 ans
Cible énergétique (kcal/kg/jour)	58	62	58	46	Equation de Schofield (poids/taille)

Table 1 : Cible énergétique recommandée chez l'enfant sévèrement agressé en fonction de son âge.

Equation de Schofield

**Dépense énergétique de repos estimée par l'équation de Schofield (kcal/jour)**	
**Filles**	
0-2 ans	16.252*P + 1023.2*(T/100) - 413.5
3-9 ans	16.97*P + 1.618*T + 371.2
10-18 ans	8.365*P + 4.65*T + 200
**Garçons**	
0-2 ans	0.167*P + 1517.4*(T/100) - 617.6
3-9 ans	19.6*P + 1.033*T +414.9
10-18 ans	16.25*P + 1.372*T + 515.5
Poids en Kg ; Taille en cm ; Résultats en Kcal/j	

Lorsque l'enfant est stable et en phase de récupération (sortie des SIP, patients chroniques), la cible énergétique devrait prendre en compte l'activité physique, la croissance et la dette énergétique cumulée. Sa dépense énergétique de repos peut être augmentée de 20-50% en fonction de la situation, notamment si l'enfant marche.

Contrairement aux besoins énergétiques, les besoins protéiques de l'enfant sévèrement agressé sont élevés en lien avec une perte urinaire d'azote augmentée. L'apport recommandé pour équilibrer son bilan azoté est de minimum **1.5 g/kg/jour**, voire davantage chez les enfants âgés de plus de 4 ans.

Les cibles caloriques et protéiques doivent être entrées dans Métavision afin de visualiser au quotidien la situation nutritionnelle de l'enfant.

Protocole d'alimentation et suivi du transit

9. INDICATIONS A L'ARRÊT DE LA NUTRITION

L'arrêt de la nutrition ne doit en principe intervenir qu'en cas de péjoration aigüe du patient:

- Hémodynamique : risque de bas débit mésentérique
- Respiratoire : risque d'intubation ou de ré-intubation potentielle
- Digestif : suspicion d'iléus ou d'entérocolite nécrosante
- Neurologique : troubles de l'état de conscience avec risques de broncho-aspiration

Il est également indispensable d'interrompre l'alimentation avant une chirurgie élective. Les délais d'arrêt d'alimentation sont définis dans la procédure SIP-PRO-0017 / Arrêt de l'alimentation lors d'une procédure d'Anesthésie.

Lorsque le patient présente une stase gastrique avec des résidus élevés >4 ml/kg chaque 4h (due par exemple à un spasme du pylore et à une motilité du tube digestif diminuée dans le contexte d'un traitement d'opiacés), il convient de :

- **Maintenir une AEDC** même si le débit ne peut pas être augmenté comme souhaité/prescrit.
- Envisager de poser une sonde nasoduodénale (y compris par les gastro-entérologues) si le patient est initialement nourri en gastrique.
- Augmenter/changer les pro-cinétiques employés (voir point 11).

10. CHOIX DU PRODUIT D'ALIMENTATION

Le choix du produit d'alimentation dépend de l'âge, du poids et de l'indication.
En raison des problèmes de transit (constipation et diarrhées) dans l'unité les produits de nutrition choisis contiennent tous des fibres.

1. Jusqu'à l'âge de 1 an : lait maternel ou lait artificiel (cf. laits artificiels à disposition dans l'institution), laits apportés par les parents, ou :
   <u>Infatrini :</u>
   <u>Indications</u> : nourrissons dénutris, à risque de dénutrition ou en cas de retard de croissance, de besoins énergétiques accrus et/ou de restriction hydrique chez les nourrissons à partir de la naissance jusqu'à un poids de 9 kg ou jusqu'à l'âge de 18 mois.
   <u>Composition</u> : 1 kcal/ml (Pour 100 ml : 101 Kcal, 2.6 g de protéines, 10.3 g de glucides, 5.4 g de lipides, 0.6 g de fibres, GOS/FOS, 305 mOsm/l) (cf. annexe pdf).

2. A partir de 1 an :
   <u>Nutrini Multifibres :</u>
   <u>Indications :</u> pour les enfants de 1 à 6 ans ou d'un poids de 8 à 20 kg. Sans gluten et sans lactose. Ne convient pas aux nourrissons, aux patients suivant un régime sans fibres et aux patients atteints de galactosémie.
   <u>Composition</u> : 1 kcal/ml (Pour 100 ml : 101 Kcal, 2.5 g de protéines, 12.5 g de glucides, 4.4 g de lipides, 0.8 g de fibres, 205 mOsm/l)

3. Dès 6 ans :
   <u>Isosource Fibres :</u>
   <u>Indications :</u> Alimentation standard physiologique destinée aux patients qui requièrent une alimentation entérale à long terme et qui sont prédisposés à la constipation.
   <u>Composition</u> : 1 kcal/ml (Pour 100 ml : 103 Kcal, 3.9 g de protéines, 13.5 g de glucides, 3.4 g de lipides, 1.5 g de fibres, 266 mOsm/l)

D'autres solutions de nutrition existent et peuvent être utilisées si le patient le nécessite après avis des spécialistes et accord du MC de garde.

11. VITAMINES ET OLIGO-ELEMENTS

*Un complément en **vitamines et oligo-éléments** doit être fourni tant que la cible calorique n'est pas atteinte.*
Aux SIP, tous les patients reçoivent un complément en vitamines (multivitamines Cernevit® par voie intraveineuse, Multibionta® ou Supradyn® par voie entérale en fonction de l'âge), oligoéléments et cholécalciférol (vitamine D).
Les oligoéléments : 1ml/kg max 10 ml/j par voie intraveineuse et 2 ml/kg max 20 ml/j par voie entérale sauf en cas d'administration de Supradyn qui en contient déjà.
Le statut en vitamine D et en zinc doit être vérifié chez les enfants à risque et une supplémentation en zinc doit être fournie aux enfants déficitaires.
En cas d'insuffisance rénale nécessitant une dialyse péritonéale, attention à l'accumulation des vitamines et oligoéléments.

12. PROCINETIQUES

Les traitement pro-cinétiques peuvent être augmentés en dose/fréquence (cf. fichier médicaments).
Le procinétique utilisé aux SIP en première intention est la dompéridone.
Le métoclopramide est utilisé en deuxième intention (en cas d'échec d'alimentation avec la dompéridone après 48h). Le métoclopramide est surtout utile en cas de suspicion de spasme du pylore.
En dernier lieu, l'érythromycine permet de stimuler la motilité du tube digestif en cas de subiléus associé aux opiacés.

13. SUIVI ET STIMULATION DU TRANSIT INTESTINAL

<u>TRAITEMENT LAXATIF</u>
Un traitement laxatif devrait être introduit au plus tard le **3ème jour** sans selles et si possible après avoir introduit une alimentation entérale stimulant la motilité de l'intestin. Il n'est pas nécessaire d'attendre que l'alimentation entérale soit complète pour introduire des laxatifs.

Les opiacés ont un puissant effet paralysant et asséchant sur tout le tube digestif. L'association opiacés-clonidine semble induire dans notre expérience une constipation souvent rebelle chez les patients nécessitant parfois l'association de plusieurs laxatifs

ou de laxatifs et de traitements de désimpaction (suppositoires de glycérine, lavements).
Chez tous les patients, mais en particulier ceux qui ont une analgo-sédation importante et l'association opiacés-clonidine, une **surveillance quotidienne** du transit est indispensable.

Les facteurs pouvant générer ou aggraver une constipation doivent être recherchés et, dans la mesure du possible, corrigés : déshydratation, immobilisation, diète pauvre en fibres, hypercalcémie, hypokaliémie, médicaments (opiacés, spasmolytiques urinaires, anticalciques, antihistaminiques H1, neuroleptiques, antidépresseurs tricycliques, fer, calcium, etc.).

Chez un enfant connu pour constipation chronique et /ou traité chroniquement par laxatifs, il est souhaitable de réintroduire son traitement dès que possible en l'absence de contre-indications.

Aux SIP **le laxatif de premier choix** est le macrogol (Movicol). L'emploi (dose, nombre de sachets, fréquence) est décrit dans le document :
Utilisation des laxatifs à l'hôpital. Bulletin d'information CPM N°1-2012 http://tribu.intranet.chuv/content-09.09.2016_10_18.pdf

L'huile de paraffine et le picosulfate peuvent être associés en cas de constipation rebelle dès **respectivement 1 an et 2 ans**.

CAVE pour la paraffine : risque de bronchoaspiration, contre- indiqué si dysphagie, gastro-parésie, reflux gastro-oesophagien important ou trouble neurologique.

Le lactulose (Duphalac, Gatinar) a été abandonné en raison du ballonnement abdominal qu'il engendrait et des complications respiratoires consécutives.
Les mucilages sont contre-indiqués chez le patient sous opiacés (risque d'impaction fécale).

DESIMPACTION :
Pour la désimpaction, des suppositoires de glycérine (Bulboid) sont utilisés et associés lorsque nécessaire à des lavements osmotiques de NaCl 0.9% avec 10 % de glycérine 10ml/kg à répéter si nécessaire. Les lavements peuvent être augmentés à 20 ml/kg sur avis des MC.

14. VALIDATION

N° de version	Date d'élaboration	Elaboré/Modifié par :	Validé par :	Date de validation
1.0	25.06.2018	Marie-Hélène Perez (MC)<br>Corinne Jotterand (diététicienne)<br>Clémence Moulet (diététicienne)<br>Jacques Cotting (MC)	GT Certification	17.07.2018
