# Supplementary material for: Impact of a Nutrition Protocol on Vitamin D Supplementation in a Pediatric Intensive Care Unit: A Retrospective Cohort Study
Source: Clin Pract. 2025 Oct 13;15(10):186. doi: 10.3390/clinpract15100186 (PMC12563859; doi:10.3390/clinpract15100186)
Supplement: Supplementary file 1 [file clinpract-15-00186-s001.zip › NP 2021 En.pdf]

Pediatric Intensive Care Unit: Enteral Feeding and Bowel Transit Monitoring Protocol

---

1. PURPOSE

This protocol outlines the steps for initiating and advancing feeding, selecting appropriate products, identifying major contraindications, and monitoring and stimulating bowel transit in critically ill pediatric patients.

The goal is to reach the caloric and protein targets no later than 5 days after the child's admission to the PICU (= Day 4 of hospitalization, with Day 0 being the admission date).

Caloric and protein targets must be assessed, adjusted, and prescribed daily on the medical order sheet and recorded in Metavision (MV).

2. SCOPE

Applicable to all medical, nursing, and assistant staff in the Pediatric Intensive Care Unit (PICU).

3. DEFINITIONS

- CEF: Continuous enteral feeding
- NG: Nasogastric
- ND: Nasoduodenal
- NJ: Nasojejunal
- PEG: Percutaneous Endoscopic Gastrostomy
- TPN: Total Parenteral Nutrition
- PPN: Partial Parenteral Nutrition
- ECC: Extracorporeal circulation
- MV: Métavision®

4. RESPONSIBILITY

Medical and nursing supervisors are responsible for implementing this protocol.

5. DOCUMENTS AND REFERENCES

References to other protocols of the unit

6. INTRODUCTION AND GENERAL RULES

Nutrition in critically ill children is a key research and clinical focus in the PICU.

Enteral feeding should be initiated as early as possible (within the first 24 hours) in the absence of contraindications and increased gradually according to the child's risk factors and tolerance.

The longer the introduction of feeding is delayed, the poorer the tolerance and the higher the protein-calorie deficit. Both undernutrition and overnutrition increase the risk of morbidity and mortality in children.

Assessment of the child's nutritional status upon admission is essential to determine the urgency of initiating feeding, the need for vitamin and trace element supplementation, and to define caloric targets.

Body weight must be monitored rigorously throughout hospitalization as soon as the clinical condition allows it. The frequency of weighing should be assessed on a case-by-case basis.

Enteral feeding is generally introduced via CEF through a NG or ND tube, and less commonly via NJ tube or PEG.

If feeding via a NG tube fails despite prokinetic treatments, a ND tube should be placed. If ND tube placement is unsuccessful, the interventional radiology or pediatric gastroenterology team must be involved within 48 hours to perform placement under radiological or endoscopic guidance.

Oral feeding is introduced as soon as the child's clinical condition allows, ensuring that nutritional needs are always met. This may include supplementing infants' breastfeeds or bottle feeds via tube, or providing either multiple bolus feeds throughout the day or a nocturnal continuous enteral feeding via tube for older children.

In the event of enteral feeding failure (after 72–96 hours, or sooner if the child's nutritional status requires it), TPN, PPN must be initiated to prevent a significant protein-calorie deficit. If the CEF is insufficient to meet protein needs, a protein supplement (Aliped®, PPN) should be considered. Primary TPN remains an exception in the PICU, except in situations where enteral feeding is not feasible (e.g., chylothorax, major digestive surgery).

Bowel transit must be closely monitored, particularly during deep analgesia-sedation, which may cause pseudo-obstruction of the intestines, potentially leading to respiratory complications that prevent extubation or necessitate re-intubation, as well as significant infectious risks. Preventive laxative therapy should be considered during heavy sedation (no later than 72 hours).

Note: Laxatives can interfere with the absorption of certain medications. Care must be taken not to administer them simultaneously.

This document deliberately does not include dosage information. For all dosages, indications, contraindications, preparations, compatibilities, etc., please refer to the medication file of the unit.

7. INITIATION, ADVANCEMENT, AND MONITORING OF FEEDING

Enteral feeding should be initiated early in critically ill children, ideally within the first 24 hours of hospitalization, unless contraindicated:

- Digestive problems as the reason for PICU admission (e.g., post-digestive surgery; observe fasting instructions prescribed by the operator)
- First night post-cardiac surgery (light meals and fluids allowed in extubated children after a short duration of cardiopulmonary bypass)
- Low cardiac output (risk of mesenteric ischemia and necrotizing enterocolitis), regardless of etiology (cardiac, septic, etc.)

The rate of enteral feeding should be increased progressively, based on the patient's tolerance and clinical condition, in order to reach the energy target by day 4 of hospitalization.

1. <u>Neonates</u>: Feeding should be introduced according to neonatal guidelines, starting at a maximum of 20 ml/kg/day, with a daily increase of up to 10-20 ml/kg/day.

   Example: A neonate weighing 3.6 kg: 20 ml/kg = 72 ml/day → maximum enteral rate of 3 ml/h.

   In cases of particular risk (e.g., hemodynamic instability, risk of mesenteric ischemia), such as during the reinitiation of feeding after cardiac surgery, AEDC should be started at 1 mL/h and increased by 1 mL/h every 24 hours initially to assess gastrointestinal tolerance. The increase in enteral feeding (frequency and volume) will depend on clinical evolution.

2. <u>Children under 10 kg</u>: Enteral feeding is introduced at a maximum rate of 1 ml/kg/h and increased every 4 to 24 hours by 1 ml/kg/h according to gastrointestinal tolerance and clinical condition.

   Example: Infant weighing 7 kg → initial maximum rate 7 ml/h.

3. <u>Children over 10 kg:</u> Enteral feeding is introduced at 5–10 ml/h and increased by 5–10 ml/h every 4 to 24 hours based on tolerance and clinical condition.

Example: Child weighing 40 kg → start at 10 ml/h, increasing by 10 ml/h every 4–8 hours until reaching 40–50 ml/h initially, depending on available fluid intake.

Note: These intakes alone do not meet the energy requirements for a 40 kg intubated and ventilated patient. Intravenous glucose infusions provide the necessary additional calories. Protein supplementation (Aliped®) should be considered if required.

8. CALORIC AND PROTEIN TARGETS

Energy and protein intakes received by patients—automatically calculated in MetaVision®—must be checked and adapted daily. The energy contribution from IV glucose, which may be significant, especially in younger children, should be included in calculations. It is essential to ensure that each day's energy and protein intake meets the patient's requirements by calculating the energy and protein balance. The cumulative balance over the hospitalization period should also be monitored.

The energy requirements of critically ill children are lower compared to those of healthy children, primarily due to the effects of mechanical ventilation, sedation, analgesia, and lack of physical activity. When a child is ventilated, sedated, and receiving analgesia, the energy target should not exceed their resting energy expenditure, which can be estimated using the Schofield predictive equation or according to the following average values:

Table 1: Recommended energy targets for critically ill children by age (kcal/kg/day)

	0–6 months	7-12 months	1–3 years	4–8 years	9–18 years
Energy target (kcal/kg/day)	58	62	58	46	Schofield Equation (weight/height)

Schofield Equation for Resting Energy Expenditure (kcal/day)

Girls

- 0–2 years: $16.252 \times P + 1023.2 \times (T/100) - 413.5$
- 3–9 years: $16.97 \times P + 1.618 \times T + 371.2$
- 10–18 years: $8.365 \times P + 4.65 \times T + 200$

Boys

- 0–2 years: $0.167 \times P + 1517.4 \times (T/100) - 617.6$
- 3–9 years: $19.6 \times P + 1.033 \times T + 414.9$
- 10–18 years: $16.25 \times P + 1.372 \times T + 515.5$

P = weight in kg; T = height in cm; results in kcal/day

Note that the most accurate method to determine the energy needs of a critically ill child is indirect calorimetry. The Schofield equation shows an acceptable correlation.

When the child is stable and in the recovery phase (post-PICU or chronic patients), the energy target should also consider physical activity, growth, and cumulative energy debt. Resting energy expenditure can be increased by 20–50% depending on the situation, particularly if the child is ambulatory.

In contrast to energy requirements, protein requirements in critically ill children are elevated due to increased urinary nitrogen loss. The recommended intake to balance the child's nitrogen balance is a minimum of 1.5 g/kg/day, up to 3 g/kg/day, and even higher for children over 4 years of age. Protein requirements are increased in cases of renal failure requiring extracorporeal clearance and major surgery, and decreased in cases of hepatic insufficiency.

Caloric and protein targets must be entered into MetaVision to allow daily monitoring of the child's nutritional status.

9. INDICATIONS FOR STOPPING FEEDING

In principle, nutrition should only be interrupted in cases of acute deterioration of the patient:

- Hemodynamic: risk of low mesenteric blood flow
- Respiratory: risk of intubation or re-intubation
- Digestive: suspicion of ileus or necrotizing enterocolitis
- Neurological: impaired consciousness with risk of bronchial aspiration

It is also essential to stop nutrition prior to elective surgery. The timing for stopping nutrition is defined in Procedure SIP-PRO-0017 / "*Stopping Nutrition for Anesthesia Procedures*."

When the patient presents with gastric stasis and high residuals, defined as >4 ml/kg every 4 hours (for example, due to pyloric spasm or decreased gastrointestinal motility in the context of opioid treatment), the following measures should be taken:

- Return the residues if they are clear and there are no clinical symptoms such as abdominal pain, nausea, or vomiting.
- Continue CEF even if the rate cannot be increased as desired/prescribed.
- Place a ND tube (if necessary, with the assistance of radiologists or gastroenterologists) if the patient was initially fed via the gastric route.
- Adjust or change the prokinetic medications being used (see section 12).

10. FEEDING PRODUCT SELECTION

The choice of feeding product depends on the child’s age, weight, and clinical indication.

Due to transit issues (constipation and diarrhea) in the unit, all selected nutritional products contain fiber whenever possible.

1. Up to 1 year of age:

    - Breast milk or formula (see infant formulas available in the institution), as well as formulas provided by the parents. Infant formulas contain fewer calories and proteins per 100 ml than the nutritional solutions administered to older children. They can be fortified, or specific diets can be prescribed when the child's clinical condition allows.

    - Infatrini®:
        - Indications: Malnourished infants, infants at risk of malnutrition, those with growth delay, increased energy needs, and/or fluid restriction; for infants from birth up to 9 kg or up to 18 months of age.
        - Composition: 1 kcal/ml (per 100 ml: 101 kcal, 2.6 g protein, 10.3 g carbohydrates, 5.4 g fat, 0.6 g fiber, GOS/FOS, 305 mOsm/l)

2. From 1 year of age:

    - Nutrini Multifibres®:
        - Indications: For children aged 1–6 years or weighing 8–20 kg. Gluten- and lactose-free. Not suitable for infants, patients on a fiber-free diet, or patients with galactosemia.
        - Composition: 1 kcal/ml (per 100 ml: 101 kcal, 2.5 g protein, 12.5 g carbohydrates, 4.4 g fat, 0.8 g fiber, 205 mOsm/l)

3. From 6 years of age:

    - Isosource Fibres®:
        - Indications: Standard physiological nutrition for patients requiring long-term enteral feeding who are predisposed to constipation.
        - Composition: 1 kcal/ml (per 100 ml: 103 kcal, 3.9 g protein, 13.5 g carbohydrates, 3.4 g fat, 1.5 g fiber, 266 mOsm/l)

Other nutritional solutions may be used if clinically indicated, following specialist consultation and approval by the attending medical consultant.

11. VITAMINS AND TRACE ELEMENTS

Vitamins

All patients receive a vitamin supplement (multivitamins administered either intravenously or enterally).

The dosage of vitamin D provided in this form is lower than current national recommendations:

- IV: 110 IU/day for patients <35 kg, and 220 IU/day for patients >35 kg
- Enteral: 444 IU/day for patients <12 years, and 200 IU/day for patients >12 years

Trace Elements

A trace element supplement should be considered early if it is unlikely that caloric targets will be reached within 5 days.

- IV: 1 ml/kg, maximum 10 ml/day
- Enteral: 2 ml/kg, maximum 20 ml/day

Vitamin D and zinc status should be checked in at-risk children, and zinc supplementation should be provided to those who are deficient.

In cases of renal insufficiency requiring peritoneal dialysis, caution is needed due to the potential accumulation of vitamins and trace elements.

12. PROKINETICS

Prokinetic agents may be used when feeding cannot be advanced as planned (e.g., in cases of high and poorly tolerated gastric residuals, nausea, or vomiting), and their dose/frequency may be increased as necessary (see medication file).

First-line prokinetic: Domperidone is the prokinetic of choice in the PICU.

Second-line prokinetic: Metoclopramide is used if domperidone fails after 48 hours. It is beneficial in cases of suspected pyloric spasm.

Third-line prokinetic: Erythromycin can be used to stimulate gastrointestinal motility in cases of subileus associated with opioid use.

⚠ Caution: These three agents may cause adverse effects such as QT prolongation, extrapyramidal symptoms, etc. (see medication file).

13. BOWEL TRANSIT MONITORING AND LAXATIVE TREATMENT

A laxative treatment should be started no later than the third day without stools, and if possible, after introducing enteral feeding that stimulates intestinal motility. It is not necessary to wait for full enteral feeding before starting laxatives.

Opioids have a strong paralytic and drying effect on the entire digestive tract. In our experience, the combination of high-dose opioids and clonidine often causes persistent constipation, sometimes requiring the use of multiple laxatives or a combination of laxatives and disimpaction treatments (glycerin suppositories, enemas).

For all patients, especially those under significant analgesia-sedation or the opioids-clonidine combination, daily monitoring of bowel movements is essential.

Factors that can cause or worsen constipation should be identified and, as far as possible, corrected, including dehydration, immobility, a low-fiber diet, hypercalcemia, hypokalemia, and certain medications (opioids, urinary spasmolytics, anticalciques, H1 antihistamines, neuroleptics, tricyclic antidepressants, iron, and calcium).

For children known to have chronic constipation or who are chronically treated with laxatives, it is recommended to resume their usual treatment as soon as possible, provided there are no contraindications.

In the PICU, the first-line laxative is macrogol (Movicol®). Its use (dose, number of sachets, frequency) is detailed in:

Utilisation des laxatifs à l'hôpital. Bulletin d'information CPM N°1-2012 http://tribu.intranet.chuv/content-09.09.2016_10_18.pdf

Paraffin oil and picosulfate can be added in cases of refractory constipation in children aged 1 year and 2 years, respectively.

Warning for paraffin oil: risk of bronchoaspiration; contraindicated in dysphagia, severe gastroparesis, significant gastroesophageal reflux, or neurological disorders.

Lactulose (Duphalac®, Gatinar®) has been discontinued due to abdominal bloating and related respiratory complications.

Mucilages are contraindicated in patients on opioids due to the risk of fecal impaction.

DISIMPACTION:

For disimpaction, glycerin suppositories (Bulboid®) are used and, when necessary, combined with osmotic enemas using 0.9% NaCl with 10% glycerin at 10 ml/kg, repeated as needed. Enema volumes can be increased to 20 ml/kg upon medical advice.

14. VALIDATION

Version No.	Date	Prepared/Modified by	Validated by	Validation Date
2.0	27.05.2020	Marie-Hélène Perez (Doctor)	Certification Working Group	02.06.2020
3.0	23.03.2021	Maria Perez Marin (Doctor)<br>Marie-Hélène Perez (Doctor)	Extended supervisory team	08.04.2021
