# Supplementary material for: Impact of a Nutrition Protocol on Vitamin D Supplementation in a Pediatric Intensive Care Unit: A Retrospective Cohort Study
Source: Clin Pract. 2025 Oct 13;15(10):186. doi: 10.3390/clinpract15100186 (PMC12563859; doi:10.3390/clinpract15100186)
Supplement: Supplementary file 1 [file clinpract-15-00186-s001.zip › NP 2021.pdf]

Protocole d'alimentation et suivi du transit

1. OBJET

Cette procédure décrit comment introduire et augmenter l'alimentation, quels produits choisir, quelles sont les contre-indications majeures à l'alimentation et comment surveiller et stimuler le transit intestinal aux SIP.
L'objectif est d'atteindre les cibles caloriques et protéiques au plus tard dans les **5 jours** après l'admission de l'enfant aux SIP (=J4 de l'hospitalisation, J0 étant la date d'admission).
Les cibles caloriques et protéiques doivent être **évaluées, adaptées et prescrites quotidiennement sur la feuille d'OM et retranscrites sur MV.**

2. DOMAINE D'APPLICATION

Le personnel médical et infirmier ainsi que les ASSC des SIP.

3. DEFINITIONS

AEDC : Alimentation entérale à débit continu
SNG : sonde nasogastrique
SND : sonde nasoduodénale
SNJ : sonde nasojéjunale
PEG : « Percutaneous Endoscopic Gastrostomy » : gastrostomie endoscopique percutanée
TPN : « Total Parenteral Nutrition » : nutrition parentérale totale
PPN : « Partial Parenteral Nutrition »: nutrition parentérale partielle
CEC : circulation extracorporelle
MV : Métavision
OM : Ordre Médical

4. RESPONSABILITES

L'application de cette procédure est sous la responsabilité des cadres médico-infirmiers.

5. DOCUMENTS ET TEXTES DE REFERENCES

Fichier médicaments.

L:\SIP\DOCUMENTS_SIP\01-SOINS\Digestif :

- SIP-PRO-0017 Arrêt de l'alimentation lors d'une procédure d'anesthésie mise à jeun
- Produits pour alimentation par sonde.pdf – Raccourci
- Raccourci vers Laits nourrissons v2

Protocole d'alimentation et suivi du transit

Utilisation des laxatifs à l'hôpital. Bulletin d'information CPM N°1-2012 http://tribu.intranet.chuv/content-09.09.2016_10_18.pdf

6. PREAMBULE ET REGLES GENERALES

Aux SIP, la nutrition de l'enfant gravement malade est un **axe de recherche** depuis de nombreuses années.
L'alimentation entérale doit être débutée dès que possible (**dans les premières 24h**) en l'absence de contre-indications et augmentée progressivement en fonction des risques et de la tolérance de l'enfant.
Plus on tarde à introduire l'alimentation, moins bien elle sera tolérée et plus le déficit protéino-calorique sera élevé ! Une sous-nutrition et une surnutrition vont toutes deux augmenter les risques de morbidité et de mortalité chez l'enfant.

L'évaluation de l'état nutritionnel de l'enfant à l'entrée est indispensable afin de juger de l'urgence à introduire une alimentation et un supplément en vitamines et en oligo-éléments et de définir les cibles caloriques.
Le poids doit être rigoureusement suivi durant toute l'hospitalisation dès que la situation clinique le permet. La fréquence de la pesée doit être évaluée au cas par cas.

L'alimentation entérale est introduite en règle générale avec un débit continu (AEDC) par SNG ou SND, et plus rarement par SNJ ou PEG.
En cas d'échec d'alimentation par SNG, malgré les traitements procinétiques, une SND doit être posée. En cas d'échec de pose de SND, l'équipe de radiologie interventionnelle ou de gastro-entérologie pédiatrique doit être impliquée (**dans les 48h**) pour une pose sous contrôle radiologique/endoscopique.
L'alimentation active per os est introduite dès que la situation clinique de l'enfant le permet et en s'assurant que les besoins nutritionnels soient toujours couverts, par exemple en complétant les tétées/biberons des nourrissons par sonde ou en administrant soit des bolus fractionnés sur la journée ou une AEDC nocturne par sonde pour les enfants plus grands.

En cas d'échec de l'alimentation entérale (**après 72h-96h**, voire plus tôt si l'état nutritionnel de l'enfant l'impose), une alimentation parentérale totale ou partielle (TPN, PPN) doit être introduite afin de limiter le déficit protéino-calorique. Si l'AEDC est loin de couvrir les besoins en protéines, un supplément de protéines par voie IV (Aliped, PPN) doit être envisagée. La TPN en première intention reste aux SIP une exception sauf dans les situations où une alimentation entérale n'est pas envisageable (chylothorax, chirurgie digestive lourde, etc.)

Le transit doit être rigoureusement surveillé, notamment lors d'analgo-sédations profondes qui peuvent mener à des pseudo-obstructions intestinales, entraînant potentiellement des problèmes respiratoires et empêchant une extubation ou motivant une ré-intubation ainsi que des risques infectieux majeurs. Un **traitement laxatif préventif** doit être envisagé lors de sédations lourdes (**au plus tard après 72h**).

Protocole d'alimentation et suivi du transit

NB : les laxatifs peuvent perturber l'absorption de certains médicaments. Attention à ne pas les administrer en même temps !

Ce document ne comporte volontairement aucune posologie. Pour toutes les posologies, indications, contre-indications, préparations, compatibilités, etc., veuillez-vous référer au fichier médicaments.

7. INTRODUCTION DE L'ALIMENTATION, AUGMENTATION ET SUIVI

L'alimentation entérale doit être débutée précocement chez l'enfant sévèrement malade, si possible dans les **premières 24h d'hospitalisation,** à moins qu'il n'y ait une contre-indication :

- Problèmes digestifs comme motif d'hospitalisation aux SIP (par exemple post chirurgie digestive, respecter les mises à jeun prescrites par l'opérateur)
- Première nuit post chirurgie cardiaque (boissons et repas léger autorisés chez l'enfant extubé après courte durée de CEC)
- Bas débit cardiaque (risque d'ischémie mésentérique et d'entérocolite nécrosante) quelle que soit l'origine (cardiaque, septique, etc…)

L'augmentation du débit de l'alimentation entérale sera progressive, en fonction de la tolérance du patient et de sa situation clinique, de manière à atteindre la cible énergétique à **J4** (dans les 5 jours) de l'hospitalisation.

1. Chez le **nouveau-né**, l'introduction de l'alimentation se fait selon les directives de néonatologie, c'est-à-dire débuter avec 20 ml/kg/jour maximum et augmentation quotidienne de 10-20 ml/kg/jour maximum :
   Exemple : nouveau-né de 3,6kgs : 20 ml/kg =72 ml/jour => débit entéral maximal de 3 ml/h.
   *En cas de risques particuliers **(instabilité hémodynamique en particulier, risque d'ischémie mésentérique)** comme lors d'une reprise de l'alimentation après une chirurgie cardiaque, l'AEDC sera débutée à 1 ml/h et augmentée de 1 ml/h chaque 24h au début afin de tester la tolérance digestive. L'augmentation du débit de nutrition entérale (fréquence et quantité) dépendra de l'évolution clinique.*

2. Chez **l'enfant de moins de 10kgs**, l'alimentation entérale est introduite au débit maximal de 1 ml/kg/heure et augmentée toutes les 4 à 24h de 1 ml/kg/h en fonction de la tolérance digestive de l'enfant et de la situation clinique.
   Exemple : nourrisson de 7 kg => 7 ml/heure comme débit maximal initialement

3. Chez **l'enfant de plus de 10kgs**, l'alimentation entérale est introduite à 5-10 ml/h et augmentée de 5-10 ml/h toutes les 4 à 24h en fonction de la tolérance et de la situation clinique.
   Exemple : Enfant de 40kgs : 10 ml/h augmenter de 10 ml/h chaque 4-8 h jusqu'à 40-50 ml/h initialement, en fonction des apports liquidiens à disposition.

Protocole d'alimentation et suivi du transit

A noter que ces apports ne permettent toujours pas de couvrir les cibles énergétiques pour un patient de 40kgs intubé et ventilé (cf. point suivant). Néanmoins, les perfusions glucosées apportent le supplément nécessaire. Si nécessaire un supplément de protéines (Aliped) doit être envisagé.

8. CIBLES CALORIQUES ET PROTEIQUES

Les apports énergétiques et protéiques reçus par les patients – calculés automatiquement sur MetaVision - doivent être vérifiés et adaptés quotidiennement. L'apport énergétique fourni par le glucose en iv, qui peut représenter un apport élevé surtout chez les petits enfants, est pris en compte dans les calculs. Il est indispensable de s'assurer au quotidien de l'adéquation entre les apports en énergie et protéines reçus par les patients et leurs besoins, en calculant la balance énergétique et protéique. La balance cumulée sur le séjour est également à vérifier.

Les besoins énergétiques de l'enfant sévèrement malade sont bas en comparaison à ceux de l'enfant en bonne santé, notamment de par les effets de la ventilation mécanique, la sédation, l'analgésie et l'absence d'activité physique. Lorsque l'enfant est ventilé, sédaté et analgésié, la cible énergétique ne devrait pas dépasser sa dépense énergétique de repos qui peut être estimée avec l'équation de prédiction de Schofield ou selon les valeurs moyennes suivantes :

	0-6 mois	7-12 mois	1-3 ans	4-8 ans	9-18 ans
Cible énergétique (kcal/kg/jour)	58	62	58	46	Equation de Schofield (poids/taille)

Table 1 : Cible énergétique recommandée chez l'enfant sévèrement agressé en fonction de son âge.

Equation de Schofield

**Dépense énergétique de repos estimée par l'équation de Schofield (kcal/jour)**	
**Filles**	
0-2 ans	16.252*P + 1023.2*(T/100) - 413.5
3-9 ans	16.97*P + 1.618*T + 371.2
10-18 ans	8.365*P + 4.65*T + 200
**Garçons**	
0-2 ans	0.167*P + 1517.4*(T/100) - 617.6

Protocole d'alimentation et suivi du transit

3-9 ans	19.6*P + 1.033*T +414.9
10-18 ans	16.25*P + 1.372*T + 515.5
Poids en Kg ; Taille en cm ; Résultats en Kcal/j	

A noter que la meilleure méthode pour connaitre les besoins énergétiques de l'enfant gravement malade est la calorimétrie indirecte. L'équation de Schofield montre une corrélation acceptable.

Lorsque l'enfant est stable et en phase de récupération (sortie des SIP, patients chroniques), la cible énergétique devrait prendre en compte l'activité physique, la croissance et la dette énergétique cumulée. Sa dépense énergétique de repos peut être augmentée de **20-50%** en fonction de la situation, notamment si l'enfant marche.

Contrairement aux besoins énergétiques, les besoins protéiques de l'enfant sévèrement agressé sont élevés en lien avec une perte urinaire d'azote augmentée. L'apport recommandé pour équilibrer son bilan azoté est de minimum **1.5 g/kg/jour, et jusqu'à 3 g/kg/jour**, voire davantage chez les enfants âgés de plus de 4 ans. Les besoins protéiques sont augmentés en cas d'insuffisance rénale sous épuration extra rénale et chirurgie majeure, et diminués en cas d'insuffisance hépatique.

Les cibles caloriques et protéiques doivent être entrées dans Métavision afin de visualiser au quotidien la situation nutritionnelle de l'enfant.

9. INDICATIONS A L'ARRET DE LA NUTRITION

L'arrêt de la nutrition ne doit en principe intervenir qu'en cas de péjoration aigüe du patient:

- Hémodynamique : risque de bas débit mésentérique
- Respiratoire : risque d'intubation ou de ré-intubation potentielle
- Digestif : suspicion d'iléus ou d'entérocolite nécrosante
- Neurologique : troubles de l'état de conscience avec risques de broncho-aspiration

Il est également indispensable d'interrompre l'alimentation avant une chirurgie élective. Les délais d'arrêt d'alimentation sont définis dans la procédure SIP-PRO-0017 / Arrêt de l'alimentation lors d'une procédure d'Anesthésie.

Lorsque le patient présente une stase gastrique avec des résidus élevés, définis comme >4 ml/kg chaque 4h (due par exemple à un spasme du pylore et à une motilité du tube digestif diminuée dans le contexte d'un traitement d'opiacés), il convient de :

- **Redonner les résidus** s'ils sont propres et qu'il n'y a pas de symptômes cliniques comme douleurs abdominales, nausée et vomissements.

Protocole d'alimentation et suivi du transit

- **Maintenir une AEDC** même si le débit ne peut pas être augmenté comme souhaité/prescrit.
- Poser une sonde nasoduodénale (si nécessaire par les radiologues/gastro-entérologues) si le patient est initialement nourri en gastrique.
- Augmenter/changer les pro-cinétiques employés (voir point 12).

10. CHOIX DU PRODUIT D'ALIMENTATION

Le choix du produit d'alimentation dépend de l'âge, du poids et de l'indication.
En raison des problèmes de transit (constipation et diarrhées) dans l'unité les produits de nutrition choisis contiennent des fibres quand c'est possible.

1. Jusqu'à l'âge de 1 an : lait maternel ou lait artificiel (cf. laits artificiels à disposition dans l'institution), laits apportés par les parents. Les laits infantiles contiennent moins de calories et de protéines par 100 ml que les solutions de nutrition administrés aux enfants plus grands. Il est possible de les enrichir (FM) ou de prescrire des diètes lorsque l'état clinique de l'enfant le permet.
   Alternative : **Infatrini :**
   Indications : nourrissons dénutris, à risque de dénutrition ou en cas de retard de croissance, de besoins énergétiques accrus et/ou de restriction hydrique chez les nourrissons à partir de la naissance jusqu'à un poids de 9 kg ou jusqu'à l'âge de 18 mois.
   Composition : 1 kcal/ml (Pour 100 ml : 101 Kcal, 2.6 g de protéines, 10.3 g de glucides, 5.4 g de lipides, 0.6 g de fibres, GOS/FOS, 305 mOsm/l) (cf. annexe pdf).

2. A partir de 1 an :
   Nutrini Multifibres :
   Indications : pour les enfants de 1 à 6 ans ou d'un poids de 8 à 20 kg. Sans gluten et sans lactose. Ne convient pas aux nourrissons, aux patients suivant un régime sans fibres et aux patients atteints de galactosémie.
   Composition : 1 kcal/ml (Pour 100 ml : 101 Kcal, 2.5 g de protéines, 12.5 g de glucides, 4.4 g de lipides, 0.8 g de fibres, 205 mOsm/l)

3. Dès 6 ans :
   Isosource Fibres :
   Indications : Alimentation standard physiologique destinée aux patients qui requièrent une alimentation entérale et qui sont prédisposés à la constipation.
   Composition : 1 kcal/ml (Pour 100 ml : 103 Kcal, 3.9 g de protéines, 13.5 g de glucides, 3.4 g de lipides, 1.5 g de fibres, 266 mOsm/l)

D'autres solutions de nutrition existent et peuvent être utilisées si le patient le nécessite après avis des spécialistes et accord du MC de garde.

Protocole d'alimentation et suivi du transit

11. VITAMINES ET OLIGO-ELEMENTS

Vitamines :
Tous les patients reçoivent un complément en vitamines (multivitamines par voie intraveineuse, ou par voie entérale).
La posologie de vitamine D administrée sous cette forme est plus faible que les recommandations nationales actuelles ; elle est de:

- IV : 110 UI/jour pour les <35 kg et 220 UI/jour pour les >35 kg
- Entéral : 444 UI/jour pour les <12 ans et 200 UI/jour pour les >12 ans.

Oligoéléments :
Un complément en **oligo-éléments** doit être considéré rapidement si la cible calorique ne peut vraisemblablement pas être atteint dans les 5 jours.

- 1ml/kg max 10 ml/j par voie intraveineuse et 2 ml/kg max 20 ml/j par voie entérale.

Le statut en vitamine D et en zinc doit être vérifié chez les enfants à risque et une supplémentation en zinc doit être fournie aux enfants déficitaires.
En cas d'insuffisance rénale nécessitant une dialyse péritonéale, attention à l'accumulation des vitamines et oligoéléments.

12. PROCINETIQUES

Les traitement pro-cinétiques peuvent être utilisés si l'alimentation ne peut pas être augmentée comme prévu (résidus en quantité abondante et mal tolérés, nausées, vomissements) et augmentés en dose/fréquence (cf. fichier médicaments).
Le procinétique utilisé aux SIP en première intention est la dompéridone.
Le métoclopramide est utilisé en deuxième intention (en cas d'échec d'alimentation avec la dompéridone après 48h). Le métoclopramide est surtout utile en cas de suspicion de spasme du pylore.
En dernier lieu, l'érythromycine permet de stimuler la motilité du tube digestif en cas de subiléus associé aux opiacés.
Attention aux effets indésirables de ces trois molécules : allongement du QT, syndrome extrapyramidal, etc. (cf. fichier médicaments).

13. SUIVI ET STIMULATION DU TRANSIT INTESTINAL

TRAITEMENT LAXATIF

Un traitement laxatif devrait être introduit au plus tard le **3ème jour** sans selles et si possible après avoir introduit une alimentation entérale stimulant la motilité de l'intestin. Il n'est pas nécessaire d'attendre que l'alimentation entérale soit complète pour introduire des laxatifs.

Les opiacés ont un puissant effet paralysant et asséchant sur tout le tube digestif. L'association opiacés-clonidine à hautes doses semble induire dans notre expérience une constipation souvent rebelle chez les patients nécessitant parfois l'association de

plusieurs laxatifs ou de laxatifs et de traitements de désimpaction (suppositoires de glycérine, lavements).
Chez tous les patients, mais en particulier ceux qui ont une analgo-sédation importante et l'association opiacés-clonidine, une **surveillance quotidienne** du transit est indispensable.

Les facteurs pouvant générer ou aggraver une constipation doivent être recherchés et, dans la mesure du possible, corrigés : déshydratation, immobilisation, diète pauvre en fibres, hypercalcémie, hypokaliémie, médicaments (opiacés, spasmolytiques urinaires, anticalciques, antihistaminiques H1, neuroleptiques, antidépresseurs tricycliques, fer, calcium, etc.).

Chez un enfant connu pour constipation chronique et /ou traité chroniquement par laxatifs, il est souhaitable de réintroduire son traitement dès que possible en l'absence de contre-indications.

Aux SIP **le laxatif de premier choix** est le macrogol (Movicol). L'emploi (dose, nombre de sachets, fréquence) est décrit dans le document :
Utilisation des laxatifs à l'hôpital. Bulletin d'information CPM N°1-2012 http://tribu.intranet.chuv/content-09.09.2016_10_18.pdf

L'huile de paraffine et le picosulfate peuvent être associés en cas de constipation rebelle dès **respectivement 1 an et 2 ans**.

CAVE pour la paraffine : risque de bronchoaspiration, contre- indiqué si dysphagie, gastro-parésie, reflux gastro-oesophagien important ou trouble neurologique.

Le lactulose (Duphalac, Gatinar) a été abandonné en raison du ballonnement abdominal qu'il engendrait et des complications respiratoires consécutives.
Les mucilages sont contre-indiqués chez le patient sous opiacés (risque d'impaction fécale).

DESIMPACTION :
Pour la désimpaction, des suppositoires de glycérine (Bulboid) sont utilisés et associés lorsque nécessaire à des lavements osmotiques de NaCl 0.9% avec 10 % de glycérine 10ml/kg à répéter si nécessaire. Les lavements peuvent être augmentés à 20 ml/kg sur avis des MC.

14. VALIDATION

N° de version	Date d'élaboration	Elaboré/Modifié par :	Validé par :	Date de validation
2.0	27.05.2020	Marie-Hélène Perez (MC)	GT Certification	02.06.2020
3.0	23.03.2021	Maria Perez Marin (CDC)<br>Marie-Hélène Perez (MC)	Equipe d'encadrement élargie SIP	08.04.2021
