# Supplementary material for: Impact of a Nutrition Protocol on Vitamin D Supplementation in a Pediatric Intensive Care Unit: A Retrospective Cohort Study
Source: Clin Pract. 2025 Oct 13;15(10):186. doi: 10.3390/clinpract15100186 (PMC12563859; doi:10.3390/clinpract15100186)
Supplement: Supplementary file 1 [file clinpract-15-00186-s001.zip › Results and statistical tests 01.10.2025.pdf]

## **Results and statistical tests**

Demographics of the two study groups

| | **Before nutrition protocol (NP) (n = 296)** | **After NP (n = 332)** | **p-value** |
|---|---|---|---|
| **Gender male/female, n (%)** | 170 (**57.43**)/126 (42.57) | 174 (**52.41**)/158 (47.59) | 0.2072 |
| **Age (years)** | 2.29 [0.72 - 7.28] | 2.64 [0.48 - 7.12] | 0.8380 |
| **Weight at admission (kg)** | 12 [7 - 21.75] | 12 [6.15 - 20.2] | 0.5020 |
| **Height at admission (m)** | 0.91 [0.68 - 1.18] | 0.9 [0.64 - 1.17] | 0.5341 |
| **Length of stay (days)** | 5.08 [3.53 - 8.93] | 5.13 [3.13 - 8.12] | 0.8310 |
| **Pediatric Index of Mortality (PIM score)** | 2.14 [1.02 - 4.41]* | 1.40 [0.79 - 3.83]** | **0.0054** |

* n = 286 (10 patients did not have a PIM score); ** n = 324 (8 patients did not have a PIM score)
Values are expressed as n (%) for categorical variables and median [IQR] for continuous variables.
The Mann–Whitney U test was used for continuous data and the Chi-square test for categorical variables.

Vitamin D administration before and after NP (IV, non-IV and enteral feeds)

| | **Before NP (n=296)** | **After NP (n=331)*** | **p-value** |
|---|---|---|---|
| **Vit D (IU/day)** | 398.01 [143.63 - 623.68] | 480.51 [271.65 - 691.04] | 0.0007 |

*331 patients post-NP because one patient has mistaken data of vitamin D received with nutrition
Mann-Whitney U test

Vitamin D supplementation before and after NP (IV and non-IV)

| | **Before NP (n=296)** | **After NP (n=332)** | **p-value** |
|---|---|---|---|
| **Vit D (IU/day)** | 288.92 [66.07 - 471.26] | 384.44 [192.94 - 507.58] | 0.0000 |

Mann-Whitney U test

Percent (%) of patients who received vitamin D supplementation (IV and non-IV)

| | **Before NP** | **After NP** | **p-value** |
|---|---|---|---|
| % | 229/296 = 77.36% | 315/332 = 94.88% | 0.000 |

Chi-square test

Time before vitamin D administration (IV and non-IV)

| | **Before NP (n=229)** | **After NP (n=315)** | **p-value** |
|---|---|---|---|
| **Time (hours)** | 40.73 [21.57 - 65.55] | 40.98 [22.98 - 64.3] | 0.9291 |

Mann-Whitney U test

Subgroup analysis of patients who did not receive vitamin D supplementation

| | **Before NP (n=67)** | **After NP (n=17)** | **p-value** |
|---|---|---|---|
| **Age (years)** | 6.94 [3.07 - 11.30] | 7.62 [4.77 - 13.01] | 0.4463 |
| **Length of stay (days)** | 3.75 [2.65 - 4.95] | 2.96 [2.62 - 4.14] | 0.1454 |

Mann-Whitney U test

Vitamin D supplementation compared with nutrition protocol (NP) recommendations.

| | **Intravenous (IV) vitamin D NP recommendation (IU/day)*** | **IV administered vitamin D (IU/day of parenteral nutrition)*** | **p-value** |
|---|---|---|---|
| **Weight at admission <= 35kg (n=26)**** | 110 | 110 [109.99 – 110] | 0.2402<br><br>Ha:median>110 with p=0.9453<br>Ha:median<110 with p=0.1719 |

| | | | |
|---|---|---|---|
| **Weight admission > 35kg (n=1)*** | 220 | 220 [220 – 220] | 1.0000<br><br>Ha:median>220 with p=1.0000<br>Ha:median<220 with p=1.0000 |
| | **Non-IV vitamin D NP recommendation (IU/day)*** | **Non-IV administered vitamin D (IU/day of stay being fed)*** | **p-value** |
| **Age <= 12 years old (n = 283)***** | 444 | 484.36 [231.67 – 576.28] | 0.2920<br><br>Ha:median>444 with p=0.0250<br>Ha:median<444 with p=0.9812 |
| **Age > 12 years old (n = 47)***** | 200 | 407.27 [459.47 – 580.31] | 0.0000<br><br>Ha:median>444 with p=0.0015<br>Ha:median<444 with p=0.9995 |

*IU/day refers to per patient. **Patients post-NP who received IV vitamin D supplementation. ***Patients post-NP who received non-IV vitamin D supplementation, nutrition excluded.
Values are presented as n for categorical variables and median [IQR] for continuous variables.
One-sided test done with sign test.
The p-values were calculated with the Wilcoxon signed-rank test.

Vitamin D supplementation compared with Swiss Federal Office of Public Health (FOPH) recommendations before and after NP implementation.

| | **FOPH recommendation (IU/day)*** | **Vitamin D supplementation (IV and non-IV) before NP (IU/day of stay)*** | **p-value** |
|---|---|---|---|
| **Age <= 1 year old (n = 91)** | 400 | 414.75 [280.11 - 496.44] | 0.5852<br><br>Ha:median>400 with p=0.2648<br>Ha:median<400 with p=0.7991 |
| **1 - 3 years old (n = 74)** | 600 | 400.92 [210.26 - 568] | 0.0000<br><br>Ha:median>600 with p=1.0000<br>Ha:median<600 with p=0.0000 |
| **Age > 3 years old (n = 131)** | 600 | 99.31 [0 - 313.02] | 0.0000<br><br>Ha:median>600 with p=1.0000<br>Ha:median<600 with p=0.0000 |
| | **FOPH recommendation (IU/day)*** | **Vitamin D supplementation (IV and non-IV) after NP (IU/day of stay)*** | **p-value** |
| **Age <= 1 year old (n = 121)** | 400 | 388.25 [201.13 – 522.75] | 0.0928 |

| | | | |
|---|---|---|---|
| | | | Ha:median>400<br>with p=0.7664<br>Ha:median<400<br>with p=0.2928 |
| **1 - 3 years old (n = 51)** | 600 | 419.68 [273.10 – 520.16] | 0.0000<br><br>Ha:median>600<br>with p=1.0000<br>Ha:median<600<br>with p=0.0000 |
| **Age > 3 years old (n = 160)** | 600 | 355.55 [149.33 – 497.86] | 0.0000<br><br>Ha:median>600<br>with p=1.0000<br>Ha:median<600<br>with p=0.0000 |

*IU/day refers to per patient.
Values are presented as n for categorical variables and median [IQR] for continuous variables.
One-sided test done with sign test.
The p-values were calculated with the Wilcoxon signed-rank test.

Vitamin D supplementation before vs after NP

| Vitamin D supplementation (IU/day of stay) | p-value |
|---|---|
| **Age <= 1 an (n = 212)** | 0.6981 |
| **1 an < Age <= 3 ans (n = 125)** | 0.6015 |
| **Age > 3ans (n = 291)** | 0.0000 |

Mann-Whitney U test
